# Rational design of cancer gene panels with OncoPaD

**DOI:** 10.1186/s13073-016-0349-1

**Published:** 2016-10-03

**Authors:** Carlota Rubio-Perez, Jordi Deu-Pons, David Tamborero, Nuria Lopez-Bigas, Abel Gonzalez-Perez

**Affiliations:** 1 Research Program on Biomedical Informatics, IMIM Hospital del Mar Medical Research Institute and Universitat Pompeu Fabra, Doctor Aiguader 88, 08003 Barcelona, Catalonia Spain; 2 Institució Catalana de Recerca i Estudis Avançats (ICREA), Passeig Lluís Companys 23, 08010 Barcelona, Spain

**Keywords:** Cancer panels, Panels cost-effectiveness, Rational design of panels, Tumor early detection, Drug profiling of tumor cohorts, Cancer driver genes, Anti-cancer drug response biomarkers

## Abstract

**Background:**

Profiling the somatic mutations of genes which may inform about tumor evolution, prognostics and treatment is becoming a standard tool in clinical oncology. Commercially available cancer gene panels rely on manually gathered cancer-related genes, in a “one-size-fits-many” solution. The design of new panels requires laborious search of literature and cancer genomics resources, with their performance on cohorts of patients difficult to estimate.

**Results:**

We present OncoPaD, to our knowledge the first tool aimed at the rational design of cancer gene panels. OncoPaD estimates the cost-effectiveness of the designed panel on a cohort of tumors and provides reports on the importance of individual mutations for tumorigenesis or therapy. With a friendly interface and intuitive input, OncoPaD suggests researchers relevant sets of genes to be included in the panel, because prior knowledge or analyses indicate that their mutations either drive tumorigenesis or function as biomarkers of drug response. OncoPaD also provides reports on the importance of individual mutations for tumorigenesis or therapy that support the interpretation of the results obtained with the designed panel. We demonstrate *in silico* that OncoPaD designed panels are more cost-effective—i.e. detect a maximum fraction of tumors in the cohort by sequencing a minimum quantity of DNA—than available panels.

**Conclusions:**

With its unique features, OncoPaD will help clinicians and researchers design tailored next-generating sequencing (NGS) panels to detect circulating tumor DNA or biopsy specimens, thereby facilitating early and accurate detection of tumors, genomics informed therapeutic decisions, patient follow-up and timely identification of resistance mechanisms to targeted agents. OncoPaD may be accessed through http://www.intogen.org/oncopad.

**Electronic supplementary material:**

The online version of this article (doi:10.1186/s13073-016-0349-1) contains supplementary material, which is available to authorized users.

## Background

Profiling somatic mutations in the coding sequence of genes that have predictive, prognostic or diagnostic value is becoming a standard tool in clinical oncology [1, 2]. Gene panels present advantages with respect to whole-exome sequencing in the clinical and translational research settings that extend beyond cost-effectiveness. For example, they possess a higher sensitivity to detect variants and are less prone to the detection of false-positive somatic mutations [3], which are key requirements if mutations detected via gene panels sequencing are going to be used to guide targeted cancer therapies or for early cancer screening via liquid biopsies [4].

Several commercial solutions are currently available to meet the growing need of cancer gene panels. All these currently available commercial and in-house cancer gene panels rely on manually gathered cancer-related genes and/or alterations that are known biomarkers of sensitivity or resistance to targeted agents, and constitute “one-size-fits-many” solutions. In both translational and basic investigation, researchers may need to design gene panels specifically tailored for particular questions (see, for example [1, 5, 6]). The design of specific panels requires laborious search of the literature and cancer genomics resources. Furthermore, whether the panel chosen comes from a commercial source or is designed by the researcher, it is very difficult to estimate its cost-effectiveness on a cohort of cancer patients.

Our previous systematic analysis of large cancer cohorts [7], which produced comprehensive catalogs of driver genes [8] across 28 cancer types, together with an in-house expert-curated compilation of tumor alterations, relevant to tumorigenesis or influencing drug effect, provide an opportunity to solve the aforementioned hurdles. Here, we present OncoPaD (http://intogen.org/oncopad), to our knowledge the first web-based tool aimed at the rational design of cancer gene panels, which dynamically estimates their cost-effectiveness to profile large cohorts of tumors of 28 cancer types.

## Methods

### Cancer cohort data

Mutational cancer data were obtained from the cohort of 6792 samples from 28 cancer types collected by Rubio-Perez and Tamborero et al. [8], see reference for details on data collection. We added a cohort of 506 chronic lymphocytic leukemias (CLL) from Puente et al. [9]

A panel can be designed to profile any of the 28 cancer types (i.e. a comprehensive solid and hematologic panel), for a group of them (e.g. a panel only for hematologic malignancies or for lung carcinomas) or for an individual cancer type (e.g. a panel for breast cancer). Additional file 1: Table S1 presents a list of all cohorts included and cohort groups pre-built in OncoPaD.

### Integrating lists of known cancer driver genes

We prepared four lists of interesting genes as input candidates for panel design:

1. The Cancer Drivers Database (http://www.intogen.org/downloads; 2014.12) [8] of genes driving tumorigenesis of cohorts of 28 cancer types.
2. The Cancer Gene Census [10].
3. Genes with validated oncogenic mutations in specific cancer types from a manual in-house compilation (see below).
4. Specific CLL (underrepresented in the cohorts in (1)) drivers from Puente et al. [9].

We integrated these four lists into a complete and reliable catalog of cancer driver genes as input of OncoPaD. Although the four lists have several genes in common, they are complementary as each of them is generated through a different approach (see Additional file 2: Supplementary Methods for more details; Additional file 3: Table S2 contains the driver genes comprised in each list).

### Prioritization of panel candidates

OncoPaD computes the cumulative mutational frequency (CMF) of the panel in the cohort of the tumor type(s) selected by the user as the number of tumors bearing protein-affecting mutations (PAMs; see Additional file 2: Supplementary Methods for details on mutations considered) in each gene (or hotspot) but with no mutations in previously considered elements:

$$ CMFite{m}_n=CMFite{m}_{n-1}+\frac{\left\{ samples withPAMs\in ite{m}_n\right\}\notin \left\{ samples withPAMs\in item{s}_{i..n-1}\right\}}{\left\{ samples\in panelcohort\right\}}\mathrm{item}:\kern.1em \mathrm{gene}\kern.2em \mathrm{or}\kern.2em \mathrm{gene}\kern.2em \mathrm{hotspot} $$

The tool also calculates two additional CMFs to compute the coverage of tumors with two or three mutations in the genes within the panel. The elements in the panel are ranked according to their contribution to the increase of the CMF. OncoPaD computes the regression line of the CMF distribution and identifies three tiers of candidate items to include in the panel (see Additional file 2: Supplementary Methods for details):

1. Tier 1 candidates: genes and/or mutational hotspots that contribute the most to the slope of the CMF distribution, i.e. to the mutational coverage of the panel.
2. Tier 2 candidates: their contribution to the CMF distribution is smaller than that of genes and/or mutational hotspots of Tier 1.
3. Tier 3 candidates: all other genes and/or mutational hotspots included in the panel. Their contribution to the coverage of the panel is negligible.

Tier 1 candidates are preferred to design the panel. Tier 2 candidates may be included if maximum coverage of the mutations in the cohort is desired, although their inclusion may reduce sequencing depth. The users may fine-tune Tier 1 candidates if they comprise a long list using the *Tier 1 stringent classification* option (see Additional file 2: Supplementary Methods).

### Identification of hotspots with high density of mutations

We designed a simple algorithm for the identification of mutational hotspots. Briefly, it iteratively identifies the minimum number of base pairs regions (of at most 100 bps) across the sequence of the gene that contain most of its mutations (see below). In each iteration the hotspot with the highest number of mutations is identified. Its mutations are then removed from the gene before the following iteration. The search stops when all sites left in the gene contain fewer than two mutations. After all hotspots are identified, the algorithm checks whether all hotspots identified account for at least a minimum fraction of all the mutations in the gene (set at 80 % by default, but configurable by the user). If this is the case, all identified hotspots are incorporated individually into the panel (see Additional file 2: Figure S1); else, the complete exome of the gene is incorporated into the panel.

### Resources used to annotate mutations and genes in the panel

To provide the designer of the panel ancillary information on relevant mutations associated to tumorigenesis or response to anti-cancer drugs we have retrieved information from the following sources (see details in Additional file 2: Supplementary Methods):

1. A list of validated oncogenic mutations, obtained from the catalog of driver mutations of Tamborero et al. (*in preparation*, available at www.intogen.org/downloads), which contains somatic and germline mutations whose role in oncogenesis has been experimentally validated in different cancer types.
2. A list of mutations known to predict sensitivity or resistance to anti-cancer drugs, obtained from the Cancer bioMarkers database by Tamborero et al. (*in preparation*, available at https://www.cancergenomeinterpreter.org/biomarkers), which contains expert curated annotations of genomics biomarkers associated to a drug effect on tumors, either drug “response” or “resistance.”

At gene level, OncoPaD adds information regarding the mode of action of the gene in cancer (i.e. a prediction on whether it acts through loss of function or activation) and the tendency of mutations in the gene to occur in the major clone in specific cancer type(s) according to the Cancer Drivers Database [8]. Data retrieved from all aforementioned resources will be continuously updated as new releases become available.

### Design and implementation of the OncoPaD web service

OncoPaD imposes no computational burden on its users beyond the employment of a reasonably modern web browser; no browser plugins are needed. The users are required to register using the Mozilla Persona service just to keep track of the visits and jobs run at the server.

The OncoPaD web service is implemented in Python 3 and relies on the CherryPy web framework [11]. The reports of the results of the panel use several Javascript resources, such as the Highcharts [12] line plots to represent the mutational coverage, and the Mutations Needle Plot [13] to represent the distribution of mutations across the protein sequence of a gene. All reports may be downloaded as a PDF file, including all charts and tables, and the genomic location of the panel candidates can be downloaded in a BED file. The complete web service implementation is available for download to academia at https://bitbucket.org/bbglab/oncopad under an ad hoc Free Source Code License Agreement.

## Results and discussion

### OncoPaD is a tool for the rational design of gene panels

**Fig. 1:**
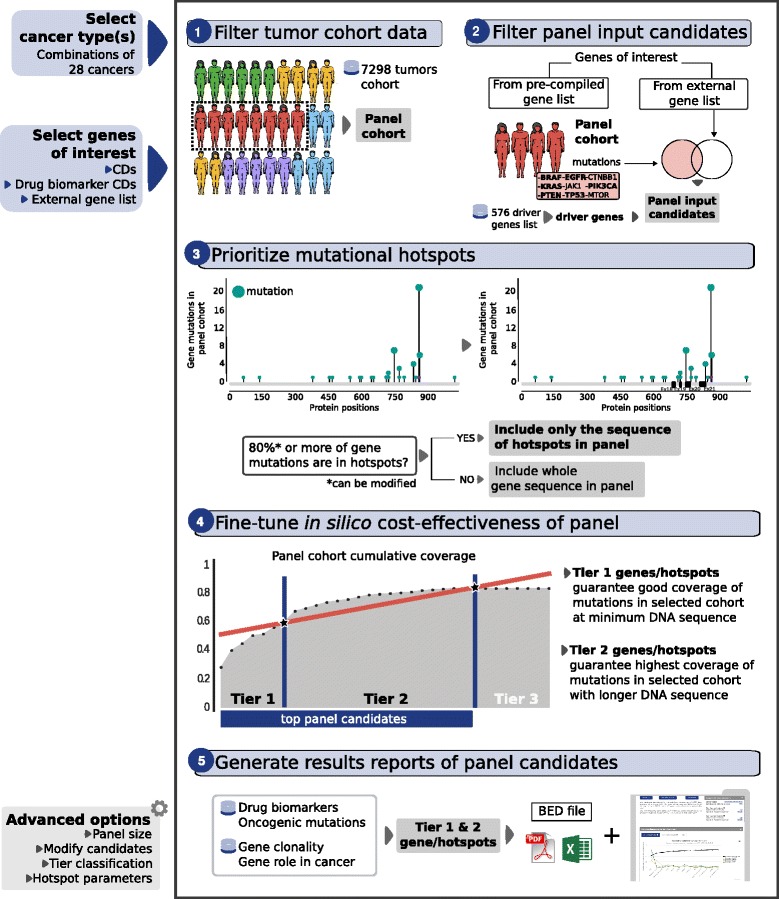
*Illustration* of the rationale of OncoPaD and its use. *Left*: Information required to start the design of a panel. It consists of two mandatory parameters: (1) cancer type(s) of the panel (*top*) and (2) genes of interest: (a) cancer driver genes (CDs), (b) CDs with drug biomarkers, or (c) a list provided by the user (*middle*). Some advanced parameters are configurable to design the panel (*bottom*). *Right*: OncoPaD algorithm. OncoPaD filters a pan-cancer cohort (7298 samples) by the cancer type(s) selected by the user (1), thus producing the cohort relevant for the panel; next, the genes relevant to tumorigenesis in the panel cohort are chosen from those selected by the user (2); the mutational hotspots of these genes are identified (details in Additional file 2: Figure S1 and the "Methods" section) (3); the cumulative distribution of mutations (or coverage) of selected genes and/or hotspots in the panel cohort is built and those that contribute the most to this coverage (Tiers 1 and 2) are selected (4); finally OncoPaD generates reports of the main features of the designed panel, with additional ancillary information of all genes and/or mutational hotspots in the panel (5)

OncoPaD builds upon systematic analyses of large tumor cohorts comprising 7298 samples [7–9, 14] to produce a comprehensive catalog of mutational drivers specific for 28 cancer types. The first input of OncoPaD is the list of (1) mutational drivers of one or more tumor types and well-known cancer genes [10], (2) manually collected driver genes bearing alterations known to influence anti-tumor drug effects (biomarkers maintained in an in-house database), or (3) user-defined genes of interest (Fig. 1). The choice of a specific tumor type(s) triggers the selection of the specific list of driver genes and a subset (panel cohort) of tumors from the initial 7298 samples pan-cancer cohort. While the list of driver genes is then employed to carry out the design of the panel, the panel cohort serves the purpose to fine-tune its cost-effectiveness (Fig. 1, panels 1, 2, and 4). OncoPaD first uses the pattern of mutations observed in the sequence of each input gene across the tumors of the panel cohort, to identify mutational hotspots that accumulate the majority of the mutations detected in the gene (Fig. 1, panel 3). If such hotspots are successfully identified (see details in “Methods”), the sequence of the gene is divided into fragments; otherwise its entire exome is included within the panel. Including mutational hotspots rather than the entire sequence of genes contributes to minimize the quantity of DNA in the panel. Next, it builds the cumulative distribution of mutations observed across tumors of the panel cohort sorting all genes and/or hotspots in the process. The shape of the resulting cumulative distribution and the ranking of genes and/or hotspots is then employed to select the ones that actually increase the fraction of mutated samples of the panel cohort that would be identified by the panel, hence coverage. Selected genes and/or hotspots are divided in two tiers depending on their contribution to this coverage. Finally, OncoPaD reports back to the researcher the list of both tiers of genes and/or hotspots, with their individual contribution to the coverage and the base pairs (bps) of DNA that each would add to the panel (Fig. 1, panel 5). The reports also include details, such as the distribution of mutations across the sequence of each item, and a trove of manually collected information of each individual mutation observed in the panel cohort, including their known oncogenic potential, or their effect on tumor response to therapies. Several elements along the design process may be fine-tuned by the user to refine the design of the panel (see details in use cases available at http://www.intogen.org/oncopad/case_studies).

To our knowledge, only three other approaches (Table 1) provide the users certain support to design cancer gene panels: (1) TEAM [15], a tool which supports the design of panels for a number of diseases based on pathogenic variants with high functional impact collected from four different databases (see Aleman et al. [15] for more details on the method); (2) the approach proposed by Martinez et al. [16] to design cancer gene panels based on recurrent non-synonymous mutations across TCGA cohorts; and (3) the DesignStudio tool by Illumina Inc. (www.illumina.com/designstudio), which determines the primers and genomic coordinates of a panel designed from user provided genes. The first unique characteristic of OncoPaD, when compared to these tools, is the possibility of basing the design of the panels on the list of drivers acting in (or biomarkers of drug response relevant to) specific tumor types. This feature renders OncoPaD designed panels uniquely suitable to screen cancer cohorts, unlike those based on methods (1) and (2), whose performance is expected to be affected because not all recurrently mutated or high impacting mutated genes are relevant for cancer development, and many oncogenic mutations are known to have a low functional impact. Furthermore, OncoPaD is the only tool that conducts the fine-tuning of the panel based on its* in silico* cost-effectiveness (see below). It is also highly configurable and the reports generated include ancillary information which guide researchers in the interpretation of the results obtained on its application to a cohort of tumor samples.

**Table 1:** Comparison of OncoPaD with other resources. Six different features are included: (1) the input genes for panel design; (2) whether the resource allows to estimate (and fine-tune) the cost-effectiveness of the designed panel; (3) whether the resource provides additional ancillary annotations for mutations included in the panel; (4) whether the tool is a web service easy to maintain, evolve and use or a static resource; (5) the type of output provided to the user; and (6) the level of customization of the panel that the user can attain

	TEAM [15]	Martinez et al. [16]	Design studio	OncoPaD
Input genes	• Genes with HIMs from COSMIC • User’s mutation list	Genes with NSMs in at least 4 % of samples in cohort 1	User’s gene list	• Driver genes in 28 cancer types • Genes with drug biomarkers • User’s gene list
*In silico* performance		Fraction of tumors from cohort 1 with NSMs	Kbps included in the panel	• Fraction of tumors from cohort 2 with PAMs • Kbps included in the panel
Metadata of panel mutations	Functional impact (SIFT and Polyphen)			• Validated oncogenic mutations • Drug biomarker mutations
Type of resource	Web service	Static panels	Web service	Web service
Output	Json file with selection of genes	List of ranked pan-cancer and per cancer type genes	• Bed file • Panel primers	• Reports with information on mutations included in the panel and performance (interactive HTML/PDF/Excel/Bed file)
User customization options	• Filter by genes with HIMs • Filter by genes found in COSMIC • Add/remove genes		Input gene list	• Cancer type(s) to design the panel • Panel input genes (pre-compiled lists of drivers/biomarkers and/or user defined). • Fine-tune the design of the panel

cohort 1: 3192 samples from ten cancer types; cohort 2: 7298 samples from 28 cancer types

*HIM* high impacting mutation, *PAM* protein-affecting mutation, *NSM* non-synonymous mutation

Note that OncoPaD, as TEAM [15] and the approach presented by Martinez et al. [16], aims to design gene panels to detect exclusively protein-coding point mutations and small indels. This is a limitation of the three methods, since copy number alterations, translocations, and non-coding mutations, which may be relevant for cancer development and the response to anti-cancer treatments, are not targeted for detection. This is the result of several decades of research on cancer overwhelmingly focused on the relevance of coding point mutations. As more information on other driver alterations—in particular arising from the analysis of tumor whole-genomes—becomes available, we will include it within OncoPaD to support the design of more comprehensive cancer gene panels.

### OncoPaD designs highly cost-effective panels

We compared the cost-effectiveness of OncoPaD designed panels to that of several available panels in three research scenarios. To carry out the comparisons, we first defined (and computed *in silico*) the cost-effectiveness of a gene panel as the balance between the fraction of samples of a cohort with mutations in genes contained in it (coverage), and the total DNA amount (Kbps). We used this *in silico* representation as a proxy of the real-life cost-effectiveness of a gene panel.

**Fig. 2:**
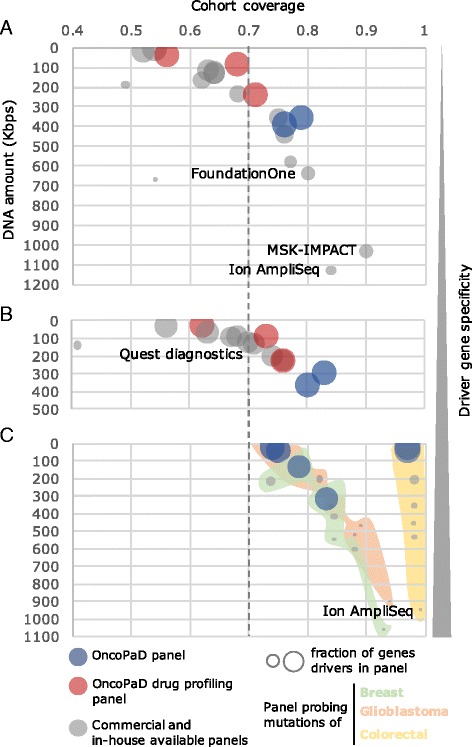
Cost-effectiveness of OncoPaD and widely employed panels. **a** Cost-effectiveness of pan-cancer panels. The *bubble plot* presents in the *x-axis* the cohort coverage of each panel—i.e. proportion of samples of the pan-cancer cohort mutated in genes and/or hotspots of the panel—versus the amount of DNA (Kbps) included in each panel (*y-axis*). The size of the bubbles represents the proportion of genes in the panel that are cancer driver genes according the four lists integrated in OncoPaD (see “Methods”). *Red bubbles* correspond to OncoPaD panels focused on drug profiling, i.e. considering as input driver genes drug biomarkers; *blue bubbles* are OncoPaD panels based on driver genes; *gray bubbles* represent other widely employed panels. **b** Cost-effectiveness of panels in the evaluation of solid tumors. **c** Cost-effectiveness of cancer type-specific panels. OncoPaD panels fine-tuned for glioblastoma (*pale green area*), breast cancer (*pale red area*), and colorectal cancer (*pale yellow area*) were built and assessed in comparison to four pan-cancer and one solid tumor-specific widely employed panels. All data on coverage and DNA amount used to build these graphs is available in Additional file 4: Table S3

We first compared the cost-effectiveness of OncoPaD panels and 13 widely-employed panels, including the *TruSight Amplicon Cancer Panel* provided by Illumina, the *Gene Read DNAseq Targeted Panels v2* from QIAGEN and the *xGen® Pan-Cancer Panel* of Integrated DNA Technologies, the only one including in its design a list of cancer driver genes [17] on a ~7000 tumors pan-cancer cohort (Fig. 2a, Additional file 4: Table S3A). In the coverage versus DNA amount space presented in Fig. 2a, the closer a panel (individual circles) is to the top right corner, the higher its coverage of mutated tumors in the cohort and the lower its content of DNA and, therefore, the higher its cost-effectiveness. For example, the *MSK-IMPACT* panel would achieve the highest coverage (90 %), but at the cost of sequencing 1030 Kbps of DNA from each sample. The *Comprehensive Cancer Panel* (Ion AmpliSeq™) and the *Pan-cancer* (FoundationOne®) panels would attain 84 % and 80 % coverage by sequencing 1130 and 634 Kbps of DNA, respectively. On the other hand, an OncoPaD designed panel for all cancer types including Tier 1 genes and hotspots would achieve 79 % coverage, but sequencing only 355 Kbps of DNA, roughly half of that sequenced by the latter and less than one-third of the former, thus with higher cost-effectiveness (blue circles). If the task at hand was the design of a panel to screen the same pan-cancer cohort for known targetable mutations (within our in-house database of biomarkers; see “Methods” for details), the highest cost-effectiveness would correspond to an OncoPaD designed panel including hotspots for drug profiling (Tiers 1 and 2), where the starting list of genes is specifically selected for mutations that influence the effect of a drug. Such a panel would cover 68 % of the pan-cancer samples sequencing only 83 Kbps of DNA (red circles).

We speculated that the cost-effectiveness of OncoPaD panels should increase the more homogeneous the cohort under screening is in terms of cancer types represented because their design relies on tumor type specific drivers. Therefore, we next compared the cost-effectiveness of OncoPaD and commercially available panels screening only the subset of solid tumors within the pan-cancer cohort (Fig. 2b, Additional file 4: Table S3B). Here, the advantage of OncoPaD panels among all those evaluated is more apparent. Specifically, an OncoPaD hotspots (Tier 1) designed panel would cover the highest fraction of solid tumors in the cohort (83 %), sequencing only 291 Kbps of DNA. To stratify solid tumors potentially responsive to anti-cancer agents, three OncoPaD designs would provide information about all tumors in the cohort, followed by the *OncoVantage Solid Tumor Mutation Analysis* (Quests diagnostics) (97 %). Finally, we compared the cost-effectiveness of panels in screening tumor type-specific cohorts (Fig. 2c, Additional file 4: Table S3C). While all assayed panels would detect between three-quarters and four-fifths of breast carcinomas, between three-quarters and nine-tenths of glioblastomas and virtually all colorectal adenocarcinomas, OncoPaD designed panels would do that by sequencing a dramatically smaller amount of DNA. For instance, the *Comprehensive Cancer Panel* (Ion AmpliSeq™) panel would cover 99 % of the tumors in the colorectal cohort, sequencing 862.21 Kbps of DNA, compared to 97 % with 21.61 Kbps of DNA (40 times less) of an OncoPAD whole genes Tier 1 panel, consequently increasing the number of samples that can be analyzed in parallel and/or increasing sequencing coverage. It is also important to bear in mind that while the genes in all OncoPaD panels are drivers of each tumor types, other panels include genes which are not implied in tumorigenesis in the tumor type(s) of the panel cohort (or any tumor type) and may lead to the detection of false positives. This would increase their likelihood of detecting false-positive mutations (either germline or somatic unrelated to tumorigenesis) [3], a feature which may turn key when the material sequenced comes from a paraffin-fixed sample with no normal DNA to filter the variants in the patient’s genome.

Additionally, we assessed the cost-effectiveness of available solid tumor panels (see above) and OncoPaD solid tumors panels on a cohort of cervical and endocervical cancer which is not currently included in the OncoPaD pan-cancer cohort (Additional file 2: Figure S2), to assess the capacity of extrapolation of the catalog of driver genes included in the tool to novel not covered cancer types. An OncoPaD panel of Tier 1 genes exhibited the highest cost-effectiveness, with the *Centrogene* panel producing a greater coverage of the tumors of the cohort, but at the expense of sequencing four times more DNA. Note that OncoPaD will be continuously updated as new sequenced tumor cohorts and lists of novel cancer driver genes and drug biomarkers become available.

In summary, OncoPaD designed panels present better cost-effectiveness than their currently available counterparts. Furthermore, the availability of several lists of genes relevant to tumorigenesis in different cancer types or specifically informative of the response to anti-cancer drugs provides them a unique versatility with respect to available one-size-fits-many solutions.

### Use case: designing a panel with OncoPaD to screen the drug response of a cohort of lung carcinomas

OncoPaD will help clinicians and researchers design NGS panels to detect circulating tumor DNA or biopsy specimens, thereby facilitating early and accurate detection of tumors, genomics informed therapeutic decisions and patient follow-up, with timely identification of resistance mechanisms to targeted agents (researchers dealing with studies as the ones exemplified in the “Background” section constitute the natural users of OncoPaD). We illustrate its use in three specific real-life research questions through tutorials available at http://www.intogen.org/oncopad/case_studies.

**Fig. 3:**
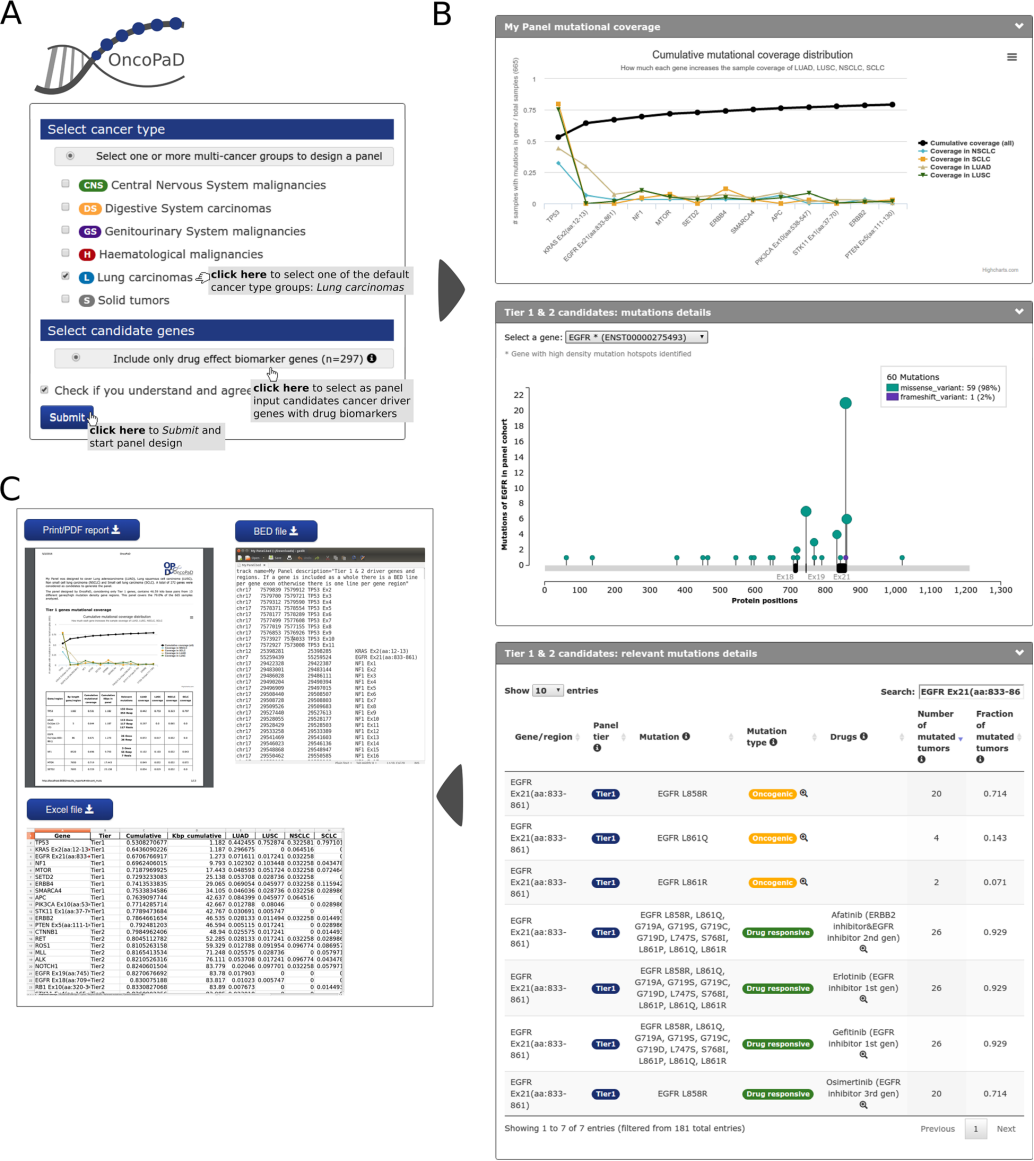
Designing a panel to screen the response to drugs of a cohort of lung carcinomas. **a** Input required by OncoPaD to design the panel. **b** Simplified *illustration* of panel reports. From *top* to *bottom*: (1) cumulative coverage of Tier 1 panel candidates in all lung carcinomas (*black line*) and coverage in each individual cohort of lung tumors included in the panel cohort (*blue*, *yellow*, *pale brown*, and *green lines*); (2) *needle plot* of the number of protein affecting mutations found along the sequence of one of Tier 1 candidates (*EGFR*) (*green* and *violet needles*), hotspots appear as *black rectangles* on the *x-axis*; and (3) annotation of drug response and oncogeneicity of gene panel mutations in the hotspot of *EGFR* exon 21. **c** Available format to download OncoPaD panel details: BED file, Excel file or PDF

In this section, we briefly present one of them, the use of OncoPaD for the design of drug screening panels for lung carcinomas. First, a subset of tumors resulting from pooling all lung carcinomas in the pan-cancer cohort is selected to compute the panel cost-effectiveness and lung cancer driver genes containing biomarkers of drug response are selected to integrate it (Fig. 3a). Upon submitting this selection, the user obtains the cumulative coverage of samples in the subset bearing mutations in the genes and/or hotspots included in the panel, sorted by their contribution (*top* panel Fig. 3b), here can observe how the panel generated covers 79 % of lung carcinomas including only 46.59 Kbps. He is also able to visualize the actual distribution of mutational hotspots in each gene in the panel. For example, as exemplified in the middle panel of Fig. 3b, two mutational hotspots of *EGFR* contribute to the panel, although only one of them is included in Tier 1. Furthermore, the bottom panel of Fig. 3b provides a glimpse at the table where the user finds further ancillary information on the relevance of individual mutations in oncogenesis or influencing drug response. Finally, the designed panel can be downloaded as a BED file, an Excel file with multiple sheets or a PDF file with the complete HTML report (Fig. 3c).

## Conclusions

We have presented OncoPaD, to our knowledge the first tool aimed at the rational design of cancer gene panels. The estimated cost-effectiveness of OncoPaD designed panels surpasses that of their currently available counterparts. The intuitive design and versatility of the tool will aid clinicians and researchers in the design of panels to address a variety of translational and basic research questions.

## Additional files

Additional file 1: Description of tumor cohorts. Excel file with description of the mutational datasets currently used by the OncoPaD web service (Table S1)﻿. (XLSX 6 kb) Additional file 2: Supplementary Data. PDF file containing the headers and legends of Tables S1–S3, Figures S1 and S2, and Supplementary Methods. (PDF 421 kb) Additional file 3: Drivers information. Excel file containing the list of driver genes per tumor type currently used by the OncoPaD web service (Table S2). (XLSX 44 kb) Additional file 4: Comparison with commercial panels. Excel file containing the results of the comparison of gene panels designed employing OncoPaD with commercially available panels (Table S3)﻿. (XLSX 34 kb)

## Abbreviations

bps: DNA base pairs
CLL: Chronic lymphocytic leukemia
CMF: Cumulative mutations frequency
NGS: Next generation sequencing
PAM: Protein-affecting mutation
TCGA: The Cancer Genome Atlas
