# Supplementary material for: Rational design of cancer gene panels with OncoPaD
Source: Genome Med. 2016 Oct 3;8:98. doi: 10.1186/s13073-016-0349-1 (PMC5047348; doi:10.1186/s13073-016-0349-1)
Supplement: Additional file 2: — Supplementary Data. PDF file containing the headers and legends of Tables S1–S3, Figures S1 and S2, and Supplementary Methods. (PDF 421 kb) [file 13073_2016_349_MOESM2_ESM.pdf]

Supplementary Data

Rational design of cancer gene panels with OncoPaD

Supplementary Tables	1
Supplementary Figures	2
Supplementary Methods	5
References	8

Supplementary Tables

Table S1. Description of cancer cohort employed by OncoPaD

(A) Cancer cohorts used to compute panel coverage, including the acronyms employed in OncoPaD tool for the 28 cancer types acronyms, their full names and the number of samples in each cohort.
(B) Characterization of the default pan-cancer cohorts available for panel design.

Table S2. Cancer drivers details

Table with the lists of cancer driver genes obtained from 4 studies and integrated in the lists of drivers employed by OncoPaD. Cancer type acronyms have their full equivalents in Table S2A.

Table S3. Comparison of the cost-effectiveness of available and OncoPaD designed panels.
Same columns are shown in all tables:

> **Genes:** Number of genes (or gene regions in last OncoPaD example) in the panel.
> **Cohort fraction:** Fraction of samples (or coverage) of the pan-cancer cohort with protein sequence affecting mutations in at least one gene or region included in the panel.
> **DNA Kbps:** Total number of kilo base pairs of all genes (or regions) in the panel (obtained by adding the length of the exons of all of them).
> **Proportion of cancer drivers:** Fraction of genes in the panel that are cancer drivers, according to the three lists of drivers included in OncoPaD (see supplementary methods).
> **Proportion of drug biomarkers:** Fraction of genes in the panel with mutations that have a known effect on anti-cancer therapies (i.e., biomarkers; see supplementary methods).

(A) Comparison of the cost-effectiveness of available and OncoPaD pan-cancer panels
(B) Comparison of the cost-effectiveness of available and OncoPaD solid tumor panels
(C) Comparison of the cost-effectiveness of five commercial and two OncoPaD panels for breast carcinomas, glioblastomas and colorectal carcinomas.

Supplementary Figures

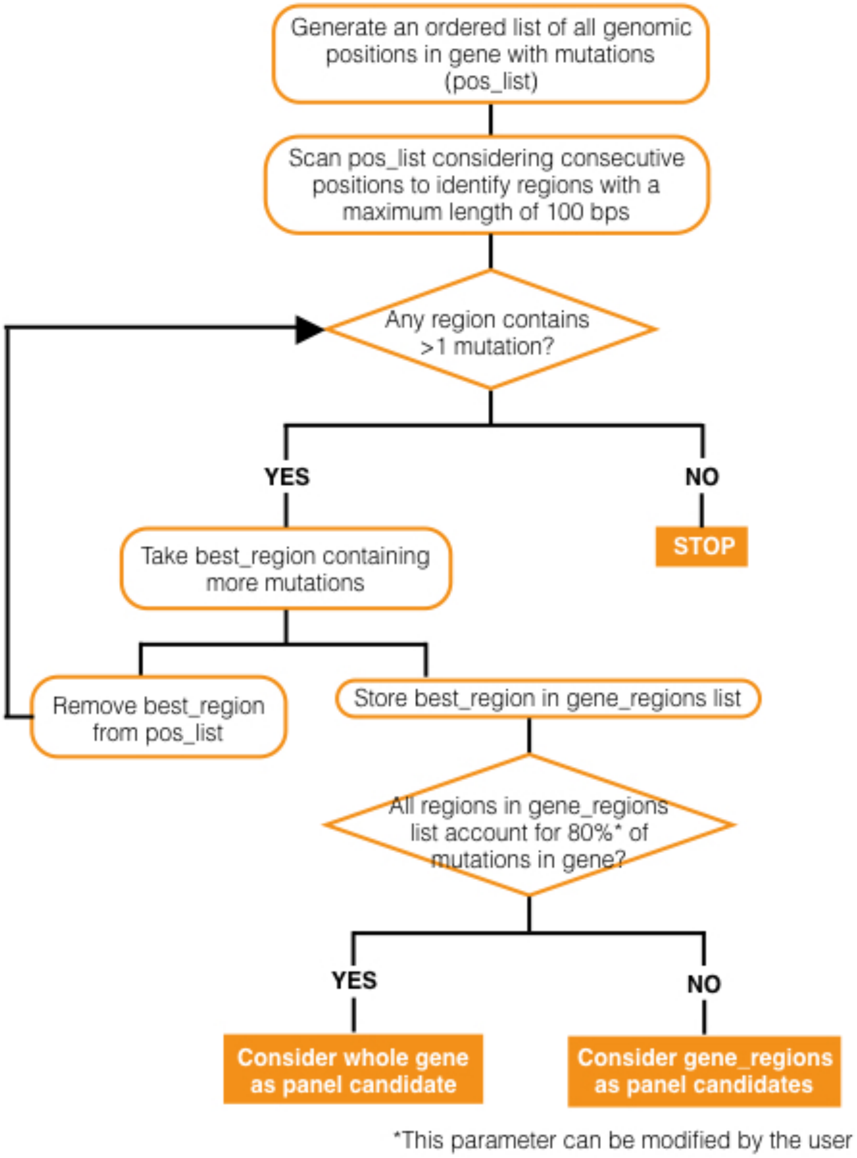

Figure S1. Decision tree of hotspot identification algorithm (see Methods).

A

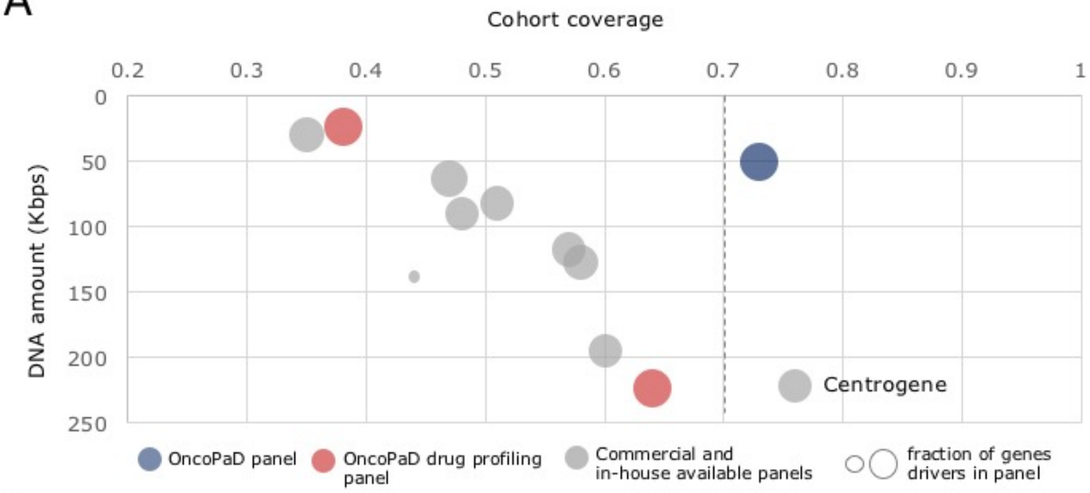

B

Panel name	Company/organization	Genes	Cohort fraction	DNA Kbps	Proportion of cancer drivers	Proportion of drug biomarkers
Solid tumor panel	Centrogene	62	0.76	222.27	0.87	0.79
SureSeq Solid Tumour Panel	Oxford gene technology	60	0.6	196.03	0.87	0.7
Solid Tumor Targeted Cancer Gene Panel	Mayo Clinic	50	0.58	127.97	0.9	0.82
Solid Tumor Mutation Panel	Arup Laboratories	47	0.57	118.1	0.89	0.79
OncoVantage Solid Tumor Mutation Analysis	Quests diagnostics	34	0.51	82.55	0.88	0.97
GeneTrails Solid Tumor	Knight labs	37	0.48	90.26	0.86	0.89
TruSight Tumor 26	Illumina Inc.	26	0.47	64.25	0.96	0.81
FusionPlex Solid Tumor Panel	Archer	53	0.44	138.4	0.32	0.42
TruSight Tumor 15	Illumina Inc.	15	0.35	30.45	0.93	0.93
**OncoPaD whole exome - drug profiling* (Tier1&2)**		64	0.64	224.38	1	1
**OncoPaD whole exome - drug profiling* (Tier1)**		10	0.38	24.18	1	1
**OncoPaD whole exome solid tumors (Tier1)**		59	0.73	51.018	1	0.54

Figure S2. Comparison of the cost-effectiveness of OncoPaD and widely employed solid tumors panels for a cervical and endocervical cancer cohort.

(A) Representation of cost-effectiveness of panels. The bubble plot presents in the x-axis the cohort coverage of each panel –i.e. proportion of samples of the cervical and endocervical cohort mutated in genes and/or hotspots of the panel– versus the amount of DNA (Kbps) included in each panel (y-axis). The size of the bubbles represents the proportion of genes in the panel that are cancer driver genes according the four lists integrated in OncoPaD (see Methods). Red bubbles correspond to OncoPaD panels focused on drug profiling –i.e. considering as input driver genes drug biomarkers–; blue bubbles are OncoPaD panels based on driver genes; gray bubbles represent other widely employed panels.
(B) Table of the cost-effectiveness of panels. Columns detailed explanation can be found in Table S1.

Supplementary Methods

Integrating lists of known cancer driver genes

We considered as input candidates for panel design the cancer driver genes identified by:

- Cancer Drivers Database (http://www.intogen.org/downloads; 2014.12). These driver genes were identified for each individual cohort from their signals of positive selection, namely, the accumulation of mutations beyond the background, their bias towards the accumulation of functional impacting mutations or, to mutations forming clusters above the background model (see Rubio-Perez and Tamborero *et al.,* 2015 for more details on the methods).

- The Cancer Gene Census. We only included genes identified through mutational evidence in any of the 28 tumor types of the pan-cancer cohort studied above.

- Included in the list of validated oncogenic mutations and annotated with a specific cancer type, genes with variants found in cancer type named *cancer* were not included in the list (see mutation annotation resources for more information on how validated oncogenic mutations list was generated).

- Puente *et al.* (2015) for CLL based on positive selection through mutation recurrence (see Puente *et al.,* 2015 for more details on the method).

The *venn diagram* on the right represent the overlap between the three first lists of cancer driver genes of all cancer types.

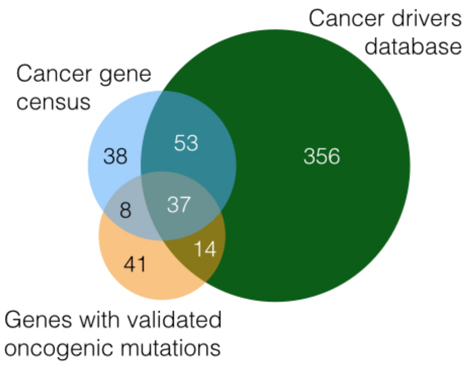

Prioritization of panel candidates

OncoPaD computes the cumulative mutational frequency (CMF) of the panel in the cohort of the tumor type(s) selected by the user as the number of tumors bearing protein affecting mutations (PAMs) in each gene (or hotspot) but with no mutations in previously considered elements. As protein affecting mutations we considered those with the following consequence types: stop gain or loss, missense and frameshift indels. Note that splice donor and acceptor consequence types are protein affecting mutations but they were excluded due to their location in non-exonic regions.

The elements in the panel are ranked following the magnitude of their contribution to the increment of the CMF. From the CMF distribution OncoPaD then infers the regression line fitting the distribution using the python *numpy polyfit* function(1) to a degree 1 polynomial. From the intersection of the regression line and CMF distribution the tool identifies 3 tiers of candidate elements to include in the panel, see figure below:

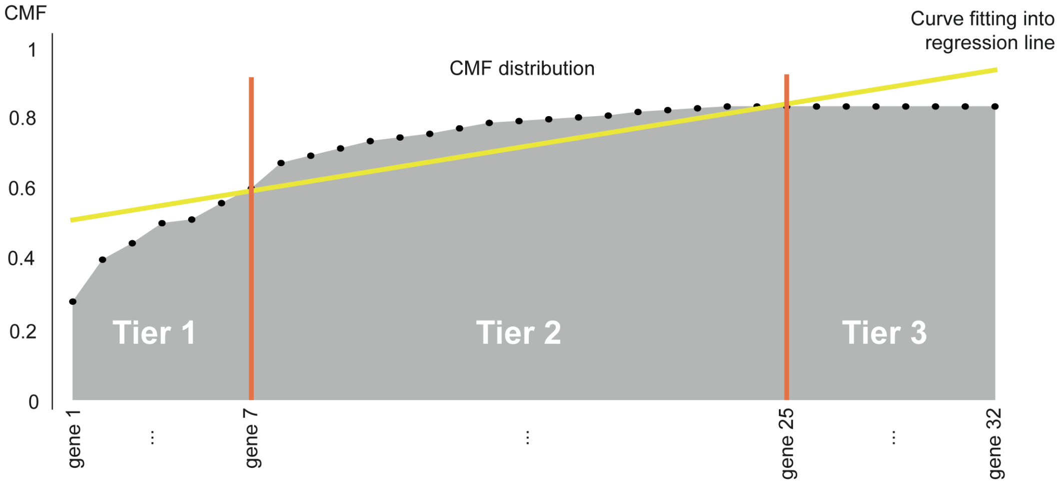

(i) Tier 1 candidates: elements located at the beginning of the CMF distribution, up to the first intersection of the regression line.

(ii) Tier 2 candidates: elements following tier 1 candidates, up to the second intersection of the regression line with the CMF distribution curve.

(iii) Tier 3 candidates: all other elements from the second intersection until the end of the CMF distribution curve.

If there are more than 5 Tier 1 candidates, they can be fine tuned by being more restrictive in the inclusion of genes in Tier 1, named **Tier 1 stringent classification**. This starts from the aforementioned classification of genes in tiers and applies the same rationale of gene prioritization through intersection of the cumulative distribution with its linear fit but based only on Tier 1 cumulative distribution. Thus, amongst Tier 1 genes it prioritizes the ones increasing more the mutational coverage, the genes between the beginning of the distribution and last intersection of the Tier 1 genes cumulative distribution, the genes after it are re-allocated as Tier 2 genes.

Resources used to annotate mutations and genes in the panel

We have retrieved information from the following sources:

a) A list of validated oncogenic mutations, obtained from Tamborero *et al.* (*in preparation*, available at http://www.intogen.org/downloads), which contains somatic and germline mutations whose role in oncogenesis has been experimentally validated in different cancer types. The list of oncogenic mutations has been culled from ClinVar(2), DoCM (http://docm.genome.wustl.edu) and Martelotto et al (2014)(3). OncoPaD only reports information on somatic mutations within this list.

b) A list of mutations known to predict drug response or resistance, integrated from the data in the Drivers Actionability Database (http://www.intogen.org/downloads; 2014.12) and Gene Drug Knowledge Database (https://www.synapse.org/#!Synapse:syn2370773). For the current version of OncoPaD we used the last merged version of both datasets from *Tamborero et al.* (*in preparation*, available at http://www.cancergenomeinterpreter.org/biomarkers). This list contains annotations for genomic biomarkers (mutations, copy number alterations, expression change and gene fusions) associated to a drug effects which have been broadly labeled as response or resistance. The information on each biomarker includes the cancer type where the drug - biomarker association has been found, along with the level of evidence of the association --i.e. whether it has been found in a clinical trial, a pre-clinical assay or reported from sporadic clinical cases. OncoPaD only reports information on mutational biomarkers. OncoPaD hotspots were mapped from genomic coordinates onto protein coordinates using CAVA(4) to associate the drug biomarkers.

At the gene level OncoPaD adds information regarding the **role of the gene in cancer** (a prediction on whether it acts through loss of function or activation). These predictions were generated for Cancer Drivers Database driver genes using OncodriveROLE(5), a random forest classifier-based tool trained with genomic data from pan-cancer TCGA cohort. Cancer driver genes from CGC were annotated as *Activating* if they were classified as *Dominant* and as *Loss of function* if they were classified as *Recessive*, ambiguous genes were classified as *No class*. OncoPaD also adds annotation on **the tendency of a gene of being clonal**, in other words, being mutated in the major clones of tumors from a certain cancer type. Major clones per cancer type were identified through pan-cancer TCGA cohort data on Variant Allele Frequency. We retrieved this information from Cancer Drivers Database too.

References

1. van der Walt S, Colbert SC, Varoquaux G. The NumPy Array: A Structure for Efficient Numerical Computation. Comput Sci Eng. 2011 Mar;13(2):22–30.
2. Landrum MJ, Lee JM, Benson M, Brown G, Chao C, Chitipiralla S, et al. ClinVar: public archive of interpretations of clinically relevant variants. Nucleic Acids Res. 2015 Nov 17;44(D1):D862–8.
3. Martelotto LG, Ng CK, De Filippo MR, Zhang Y, Piscuoglio S, Lim RS, et al. Benchmarking mutation effect prediction algorithms using functionally validated cancer-related missense mutations. Genome Biol. BioMed Central Ltd; 2014 Jan 28;15(10):484.
4. Münz M, Ruark E, Renwick A, Ramsay E, Clarke M, Mahamdallie S, et al. CSN and CAVA: variant annotation tools for rapid, robust next-generation sequencing analysis in the clinical setting. Genome Med. 2015 Jan;7(1):76.
5. Schroeder MP, Rubio-Perez C, Tamborero D, Gonzalez-Perez A, Lopez-Bigas N. OncodriveROLE classifies cancer driver genes in loss of function and activating mode of action. Bioinformatics. 2014 Sep 1;30(17):i549–55.
